# Inside the History of Italian Coloring Industries: An Investigation of ACNA Dyes through a Novel Analytical Protocol for Synthetic Dye Extraction and Characterization

**DOI:** 10.3390/molecules28145331

**Published:** 2023-07-11

**Authors:** Ilaria Serafini, Kathryn Raeburn McClure, Alessandro Ciccola, Flaminia Vincenti, Adele Bosi, Greta Peruzzi, Camilla Montesano, Manuel Sergi, Gabriele Favero, Roberta Curini

**Affiliations:** 1 Department of Chemistry, Sapienza University of Rome, P. le Aldo Moro 5, 00185 Rome, Italy; k.mcclure.1@research.gla.ac.uk (K.R.M.); alessandro.ciccola@uniroma1.it (A.C.); flaminia.vincenti@uniroma1.it (F.V.); adele.bosi@uniroma1.it (A.B.); peruzzi.1957090@studenti.uniroma1.it (G.P.); camilla.montesano@uniroma1.it (C.M.); manuel.sergi@uniroma1.it (M.S.); roberta.curini@uniroma1.it (R.C.); 2 Department of Earth Sciences, Sapienza University of Rome, P. le Aldo Moro 5, 00185 Rome, Italy; 3 Department of Environmental Biology, Sapienza University of Rome, P. le Aldo Moro 5, 00185 Rome, Italy; gabriele.favero@uniroma1.it

**Keywords:** synthetic dyes, ACNA, ammonia extraction protocol, dLLME, HPLC-HRMS, cultural heritage

## Abstract

The introduction of synthetic dyes completely changed the industrial production and use of colorants for art materials. From the synthesis of the first synthetic dye, mauveine, in 1856 until today, artists have enjoyed a wider range of colors and selection of chemical properties than was ever available before. However, the introduction of synthetic dyes introduced a wider variety and increased the complexity of the chemical structures of marketed dyes. This work looks towards the analysis of synthetically dyed objects in heritage collections, applying an extraction protocol based on the use of ammonia, which is considered favorable for natural anthraquinone dyes but has never before been applied to acid synthetic dyes. This work also presents an innovative cleanup step based on the use of an ion pair dispersive liquid–liquid microextraction for the purification and preconcentration of historical synthetic dyes before analysis. This approach was adapted from food science analysis and is applied to synthetic dyes in heritage science for the first time in this paper. The results showed adequate recovery of analytes and allowed for the ammonia-based extraction method to be applied successfully to 15 samples of suspected azo dyes from the Azienda Coloranti Nazionali e Affini (ACNA) synthetic dye collection, identified through untargeted HPLC-HRMS analyses.

## 1. Introduction

Historians and conservators consider 1856 a key year in the history of industry. The work of the young British chemist William Henry Perkin introduced mauveine (the first synthetic dye) [1], and this discovery started a new era in fabric dyeing. The synthesis of dyes in laboratory environments opened up the possibility of exploring new methodologies to obtain new hues and shades, which no longer relied upon the intrinsic variability of natural dye resources [2]. For this reason, the price of production was reduced and the dependency of the textile industry on natural dyes declined [3,4,5]. This synthesis completely changed the face of the industry and allowed for rapid scaling up of textile dye production and an increase in market access to textile dyes, leading to the launch of new large chemical plants and companies across the world [6,7]. 

Feverish experimentation, encouraged by the new potential in terms of possible substances, colors and synthesis routes was the result of this new phase, which invested not only the world of industry but also the world of art materials. Colored objects originating from this period of rapid expansion offer important information that can shape our understanding of the histories of industries and art that shaped the development, use and production of these materials.

This importance explains why synthetic dye studies, which represent a relatively new field in cultural heritage analyses due to their modernity, are rapidly increasing in importance, taking into account that the artistic productions of the 19th–20th century now require conservation interventions and raise curatorial questions [2,8]. 

While scientific analysis of synthetically dyed heritage objects is carried out in a similar way to the analysis of natural dyes, chemical variabilities between the two groups introduce different complexities. In general, synthetic dyes possess greater molecular uniformity than natural dyes, which are often a mixture of different chromophore compounds (i.e., we can count 68 anthraquinone dye molecules for madder roots). This is because they are produced under controlled conditions in laboratory environments, whilst natural dyes are influenced by a significant number of “uncontrolled” natural variables [4,5]. In contrast, however, a vast range of molecular classes exist for synthetic textile dyes compared to natural dyes. This reflects the extensive variability of the dye molecules able to be synthesized in the laboratory compared to those that are derived from the natural world [6]. Due to this incredibly high diversity in chemical structure, substituents, etc., it is difficult to develop analytical protocols that are well suited to the identification of all of the different chemical classes and typologies of synthetic dye; for example, charged dyes (e.g., reactive dyes) may require different extraction methods than uncharged dyes.

This variability means that different processes may be required for the extraction of different types of dyes from fibers. Furthermore, the variability introduces additional challenges for the heritage scientist in identifying the chemical structures of unknown dyes, as reference spectra do not exist for the thousands of potential commercial dyes sold, and minor variations (e.g., substituent position) make even mass spectrometry data, which do not require reference data, difficult to interpret. Reference spectra databases currently only contain a small proportion of the dyes made and sold on the market, and the vast range of available dyes make it difficult to develop, navigate and update databases—making it challenging to use techniques that rely upon comparison with known compounds. Moreover, inconsistencies in nomenclature—where different manufacturers refer to the same dye molecules by their own brand names, or use similar names for chemically different dyes—make it challenging to identify dyes even when their commercial names are listed [2,9,10]. Together, these factors make the identification of these types of dyes from historical and artistic matrices a highly complex matter; for this reason, it is desirable to develop ad hoc methodologies and (re-)organize bibliographical sources. 

However, the improved molecular uniformity within a single sample of synthetic dyes makes some aspects of analysis simpler than for natural dyes, which are generally made up of several low-concentration chromophores. For example, in spectroscopic analysis such as Raman spectroscopy, synthetic dyes often produce more intense spectral peaks. This can allow for spectra to be obtained without enhancements such as surface-enhanced Raman spectroscopy, which is required for natural dyes [2,9]. 

The development of new protocols for synthetic dye analysis represents a relatively new research area that has the potential to lead to significant improvements in how we research and understand modern heritage objects. Toward this goal, some interesting work has been published in recent years. For example, the excellent potential of nondestructive or minimally invasive analyses using Raman and SERS for providing new information on synthetically dyed heritage materials has been researched [9,11,12,13,14].

High-pressure liquid chromatography, coupled with mass spectrometry analyses (HPLC-MS)—generally considered the “gold-standard for dye analysis”—require extraction of the dye from a sample of the object. The extraction methods used for synthetic dyes are generally adapted from natural dye studies, such as oxalic acid or organic solvent, such as pyridine, at high temperatures [3,15], and applied directly to synthetically dyed textiles [12]. However, if the chemistries of the synthetic dyes under analysis are considered, more effective extraction protocols can be developed for specific objects. Furthermore, unlike other fields that employ synthetic dye analysis (e.g., food science) current methods in heritage science do not usually consider the use of cleanup steps, which are used to purify the sample before analysis. This lack of purification introduces impurities to the MS spectra that can at times produce signals that are higher than dyes or overlap their signals. These issues are even more pronounced in high-resolution mass spectrometry, which is a technique the field is increasingly moving toward. Furthermore, as synthetic dyes come from multistep synthesis and, therefore, may contain various side products, performing any separation or purification on the final compounds may not be commercially feasible for industries. Therefore, commercial synthetic dyes commonly contain a medium-high purity, and the remainder comprises side products [16]. For these reasons, the application of well-suited extraction methods (decided upon by the historical context and literary information available for the object under analysis), the use of an effective cleanup step (which isolates and purifies the molecules of interest before analysis), and analysis with high-sensitivity techniques such as UHPLC-MS is a beneficial approach that avoids sample losses, decreases spectral interference, and maximizes signal intensity [17].

Furthermore, historical textiles have occasionally been subjected to more than one dyeing process, sometimes both natural and synthetic, as demonstrated previously [18]. In these cases, two separate samples must be taken from a textile artefact that is likely to contain both natural and synthetic dyes. These factors work in opposition to the cultural heritage goal of achieving minimal destructiveness and maximum information. Recent papers are therefore starting to present methodologies that look at both components [12,19], but these methods still employ acid conditions or organic solvents at high temperatures, which have been demonstrated to be less effective in preserving the molecular pattern of natural dyes (e.g., madder, cochineal dyes) compared to ammonia methodologies [20,21].

For this reason, in this paper, the authors propose a new extraction protocol and novel clean up strategy for the recovery of synthetic dyes, starting from the innovative application of the ammonia–EDTA extraction methodology to such compounds. In particular, the method is focused on the development of a cleanup protocol to be used in the extraction of acid dyes, starting from the azo class specifically. This class of dyes, characterized by a N=N bond, was one of the earliest developed, with the first dye, Bismarck brown, commercially synthesized after 1861, shortly after mauveine synthesis [2,22]. Different colors can be obtained by modifications to the chemical structure, but the azo class is most typically associated with red, yellow and orange hues [22]. Since their first appearance on the market, azo dyes have been widely used in historical objects, representing one of the largest classes of synthetic dyes; it is therefore common to find them in early synthetic dye collections [22]. For this reason, the authors considered them a relevant class with which to start research with this specific focus. 

Several previous analytical methodologies for azo dyes have been investigated in the field of food science, as azo dyes were largely employed, such as in edible products, until the emergence of scientific evidence related to the carcinogenic effects of some compounds in this class [23]. In response, several governments banned their use and a rapid development in analytical methods for the detection and identification of azo dyes in foods became necessary for enforcement of these regulations [24,25,26]. Despite the extensive study of these dyes in food science, heritage science has not yet utilized this significant body of work in improving its methods for the identification of azo dyes.

This work therefore uses the methods developed for food science as a springboard to propose a new methodology suited to the analysis of synthetic dyes in the heritage field. The method was developed using three specific azo dyes, broadly representative of the azoic acid dye class. The standards chosen were Acid Yellow 25 (CI: 18835), Congo Red (CI: 22120) and Red 2G (CI: 18050). The choice of these three dyes lies not only in their chemical properties (good water solubility and possessing negative charges), which allows for the use of the ammonia extraction, but also because they are potential reference standards for the historical samples chosen as a case study to evaluate the effectiveness of the methodology in the art–historical context. These historical samples were taken from the Azienda Coloranti Nazionali e Affini (ACNA) collection at the Museum of Chemistry, Sapienza University, and the links to these compounds were predicted through literature research into the commercial names listed beside the samples selected for analysis.

The Museum of Chemistry, located within Sapienza University of Rome’s Department of Chemistry, holds an extensive collection of early synthetic dyes from several different dye companies and a significant group is represented by the dyes from the ACNA—an Italian chemical company active from 1882 until 1999 [7]. The collection of dyes analyzed contains dye powders in glass jars and card-backed sheets holding samples of dyed wool fibers. These samples came directly from the ACNA laboratories, and this collection is likely to date from the 1930s. 

Researching this type of collection represents a precious opportunity to deepen historical knowledge about the production of dyes in a precise historical period, which is still understudied. The data produced also contributes to the published reference spectra available for other researchers studying unknown dyes. Moreover, samples coming from the ACNA laboratories can provide useful information to reconstruct the synthetic processes followed and history of the industrial process. The nature of these samples also means that many of these dyes may have been synthesized but then discarded due to their performance on textiles not considered suitable for large-scale production or due to a lack of chemical–physical properties.

## 2. Results and Discussion

The ammonia–EDTA protocol represents the first method of dye extraction in a basic environment at room temperature. It was initially developed for natural anthraquinone dyes due to the sensitivity of many dye components to the acid environment. The method showed that, even in comparison with organic solvent methods, it could better preserve highly sensitive glycosylated moieties [20,21]. Never before applied to synthetic dyes, the present work arises from a desire to evaluate the performance of this methodology for a different dye group. One motivation for the development of a method applicable to both dyes is that it is not unusual to find textile artifacts that contain both natural and synthetic dyes, especially in the years immediately following the synthesis of mauveine [18]. In cases such as these, if this protocol was found to be applicable to synthetic dyes, it would be possible to minimize the quantity of materials and maximize the information obtained from a single extraction. 

Recently, the ammonia protocol has been successfully applied for the microgel extraction of natural anthraquinone dyes [27]. This research found that the application of a cleanup protocol strongly improves the quality of the spectra obtained, as mentioned in the introduction. Traditional liquid–liquid extraction (LLE) approaches cannot be used for the cleanup of azoic acid dyes, which are charged species stored as powders with cationic counter-ions before application to textiles to which they bind directly through their ionic group [28]. When extracted from the textiles, they revert to their anionic form and hence have a high affinity for water. This charged characteristic means they have an extremely low affinity for less polar solvents; so, it is unlikely for them to be recovered from an organic extracting solvent during liquid–liquid extraction. This is a significant gap in the literature that this paper addresses through the application of dLLME with the addition of an ion pair reagent (IP-dLLME). 

### 2.1. Development of IP-dLLME Protocol

For the development of a cleanup protocol for synthetic dyes, solvent ratios were decided by referring to a study on dLLME extractions of azo dyes from ice cream samples presented by Faraji et al. [24]. dLLME has recently been applied for the first time to heritage dye analysis, and this represents one of the first well-suited cleanup methods applied in this field [29]. The method was first developed in 2006 for environmental science [30], and nowadays is widely used in analytical chemistry fields including forensics, food science and environmental biology [30,31]. Based on a three-solvent system, dLLME involves the rapid injection of an organic extracting solvent and a disperser solvent into an aqueous solution. This forms a cloudy solution that maximizes the contact between phases, increasing the opportunities for analytes of interest to move into the extracting phase. This promotion of extraction means that smaller quantities of extraction solvent can be used with improved recoveries. This has the benefit of combining purification and extraction into a single step [29,30,31,32,33]. For synthetic azo dyes, the aim of using an ion pair reagent is to overcome their very high affinity for water and allow for transition to a less polar extracting phase during liquid–liquid extractions. The ion pair reagent tetra-n-butylammonium bromide (TBAB) was used. The quaternary character of TBAB means that it has significant steric hindrance and through this can form an “ion associate” with anionic molecules—such as azoic acid dyes [34]. These ion associate pairs are bound by the steric effects of the TBAB, and act in a similar way to nonpolar molecules. This apolar behavior allows the ion associate pair to transition into the organic extraction solvent. The use of this ion pair reagent enables the application of the dLLME cleanup protocol to synthetic dyes, and whilst it can also be used with traditional LLE, the use of dLLME enhances extraction recovery, efficiency and precision. TBAB was specifically chosen, as it is reported to be significantly more efficient than the chloride and iodide countered quaternary ammonium salts [35]. It was also successfully applied to azo dyes from food samples by Faraji et al. [24] in the protocol used as the basis for this research. 

**Figure 1:**
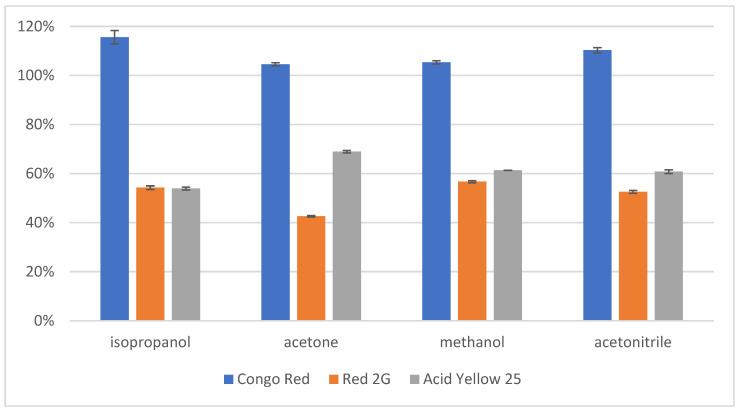
Comparison of the recoveries of three azo dye standards after the performance of dLLME with chloroform as the extracting solvent trialing four dispersers: isopropanol, acetone, methanol and acetonitrile.

The results of the disperser tests for synthetic dyes are displayed in Figure 1.

Under the IP-dLLME protocol, all dispersers display good recoveries for all analytes. The recovery of Congo Red—over 100% for all dispersers—is explained by a combination of the error margins, and the matrix effect of the TBAB ion pair reagent. Despite the errors in recovery attributed to matrix effects, the disperser trials were sufficient to evaluate that the best recovery was achieved with methanol as the disperser. Methanol was therefore used as the disperser for further tests.

To counter the matrix effects, further analyses used a spiked sample as a reference—which takes into account the matrix. The spiked sample was prepared identically to the experimental conditions described but using 100 μL methanol instead of 500 ppb reference mix. After drying, the residue was reconstituted with the 500 ppb reference mix before analysis.

**Figure 2:**
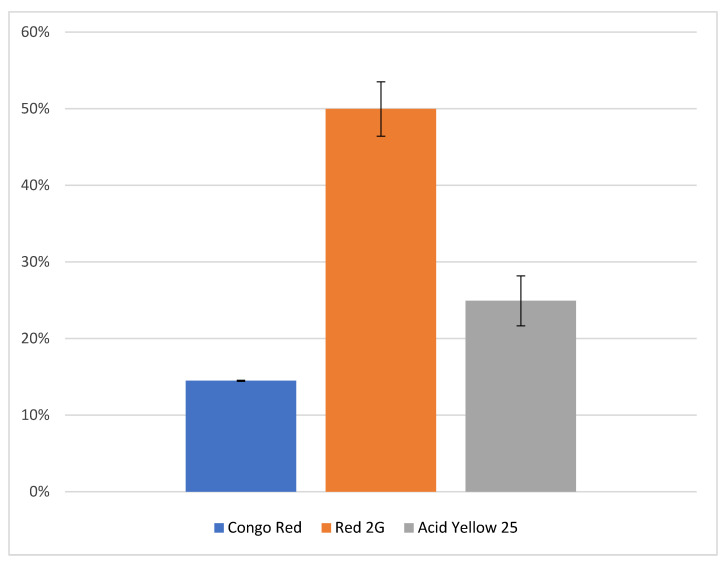
Recoveries of the three trialed synthetic dye analytes with the complete analytical protocol combining the ammonia and Na2EDTA extraction method with the novel dLLME cleanup protocol developed in this research.

After deciding the best disperser conditions for the extraction of the synthetic azo dyes, the state-of-the-art ammonia–EDTA protocol was applied alongside dLLME to assess its effects on the recovery of the samples. The results of this analysis are presented in Figure 2.

These results display a more significant reduction in recovery than was observed when dLLME was performed alone—particularly with reference to Congo Red. This reduction is likely to be attributed to the reference in this trial accounting for the matrix effect, but also could be related to the application of the extraction protocol. However, despite this reduction, recoveries are sufficient to verify the coherence and effectiveness of the complete analytical protocol. This means that the ammonia–EDTA extraction method can now be applied to synthetic dyes as well as natural dyes and brings together a coherent extraction methodology that requires only one sample to be taken from artefacts suspected to contain both natural and synthetic dyes. While the methodologies for cleanup for natural and synthetic dyes are divergent, the improved recoveries of the ammonia–EDTA extraction method are sufficient to allow for the extracted sample to be divided into two when both natural and synthetic dyes are extracted.

### 2.2. Application to the Case Study: ACNA Industries Samples

This study marks a first step toward understanding the full collection of synthetic dyes held by the Museum of Chemistry and provides insight into the naming conventions of the ACNA, an understudied dye manufacturer. Information on ACNA dye naming conventions provides a useful understanding that could aid further studies related to their dyestuffs, both within the Museum of Chemistry and elsewhere. The study of these dyes is an excellent opportunity to understand the behaviors and synthetic procedures of a company that was active throughout the 20th century. Studying this through the lens of a collection obtained directly from the manufacturer, such as the collection in Sapienza University’s Museum of Chemistry, is uniquely useful for two reasons: It offers the chance to obtain information about dyes that were available on the market—which have the potential to offer a synthetic dye database that can be referred to in the analysis of unknown objects. It also provides the opportunity to study dyes that may never have been placed on the market—which could provide insight into the internal testing methods and motivations of the ACNA. In particular, results from dyes such as Rosso Amidonaftolo 2G and Giallo Luce Solido 2G for which both powders and dyed fibers were analyzed provides interesting information regarding the consistency of naming.

The samples were first studied using nondestructive Raman spectroscopy before proceeding to extraction [8] and untargeted HPLC-HRMS analysis, presented in this paper. These results were combined to obtain information regarding the identities of the dyes studied.

**Figure 3:**
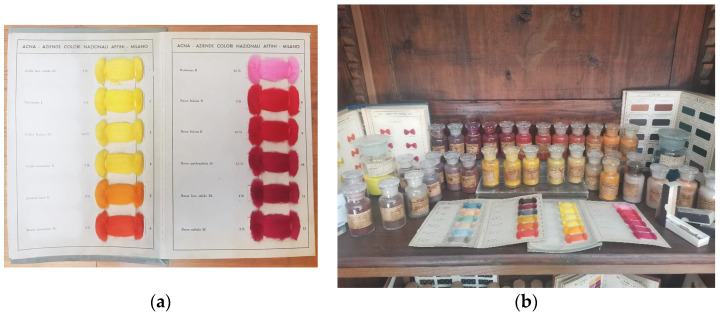
(**a**) Photograph of the card-backed sheet containing the sampled dyes. Note: all dyes were sampled except Rodamina B, which was excluded due to predictions from the name that it was likely to be a Rhodamine basic dye and hence would not be suitable for the new methodology; (**b**) photograph of part of the collection of the dyes on display from the ACNA synthetic dye collection based at Sapienza University of Rome.

Fifteen samples were taken from the collection—11 fiber samples from one card-backed sheet (Figure 3a), and 4 powder samples from glass jars (Figure 3b: photo of a part of the collection, where the jars are visible too). The samples were chosen after an initial visit, during which names were recorded to allow for literary research into their commercial names. This particular group of dyes was then chosen due to research indicating a high likelihood that a majority of the dyes in the group are of the azo class.

### 2.3. HRMS Analyses—Analytical Challenges

To pursue the scope of identification of the dye compounds presents on the museum samples, untargeted mass analysis was performed using an Orbitrap high-resolution mass spectrometer. The mass spectrometry results contained several peaks that were present in all analytes and hence likely to be related to the matrix and these were therefore discounted when analyzing the data.

Where assignments agreed with the predictions formulated from the Raman spectroscopy interpretations [9], it was concluded that the molecules were highly likely to correspond to the projected molecular structures. In cases where Raman spectroscopy was not sufficient to obtain formal predictions about the molecular structures (particularly when there were no corresponding spectra available in the literature) but the mass analyses were able to provide possible predictions, Raman spectroscopy was utilized as further confirmation of possible assignments. In some cases, characterization of the specific species was not obtained and will require additional research. 

The reasons for the difficulties associated with this characterization are the lack of databases available for the identification of synthetic dye molecules, and sometimes the dyes come from one specific company and therefore may not have been widely used or indeed ever been commercially available. Further challenges with the mass spectrometry of azoic acid dyes most likely to be present in this collection are the fact that these species contain varying degrees of charged character, meaning that *m*/*z* ratios may refer to several different molecular masses.

Alongside this, where databases exist, they generally include the counter-ion mass when reporting the overall molecular mass, so possible variations in the cationic species must be considered. A summary table (Table 1) of evidence found and/or hypothesized is presented and a case-by-case discussion, also in connection with the Raman data discussed in [9], immediately follows.

- Assignments of “Arancio Luce G”

**Figure 4:**
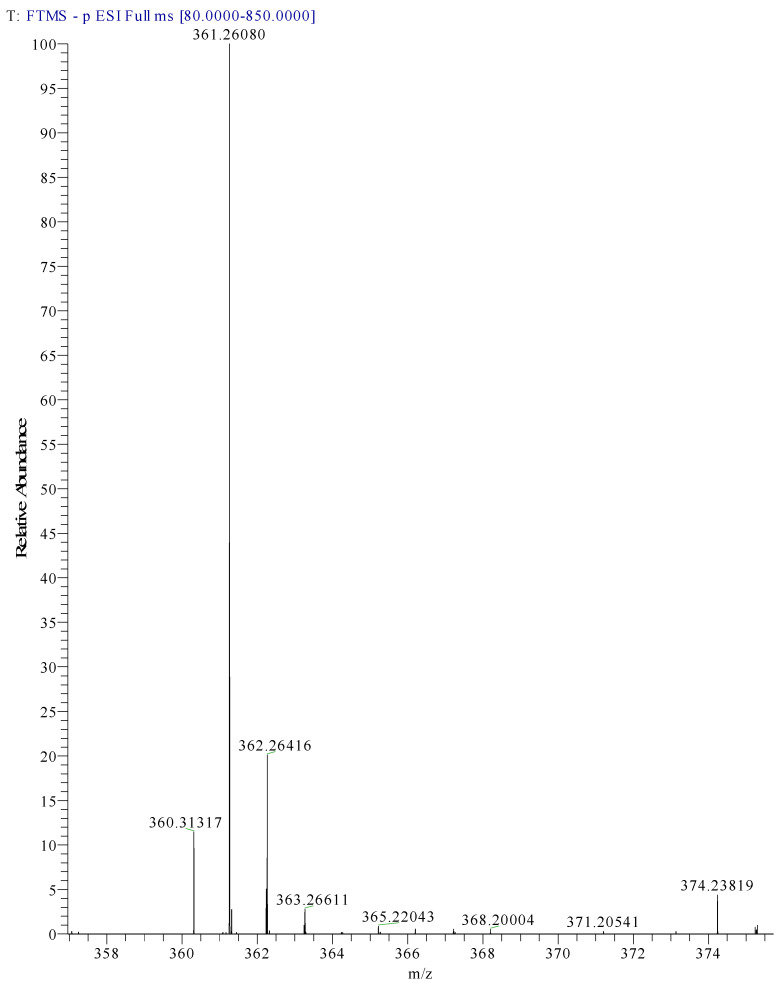
Diagnostic peak at 361.2608 from Arancio Luce G.

Preliminary research for “Arancio Luce G” suggested a possible association of this name to Acid Orange 10 and Food Orange 4, which have the same chemical structure (C.I. 16230) [8,36]. Mass spectral data of the compound were fairly weak, with three unique *m*/*z* peaks observed, all of which had relatively low intensity chromatographic peaks. The peaks observed were for *m*/*z* 360.3131, 361.2608 and 526.0877. The highest intensity of these peaks was observed at *m*/*z* 361.2608 and this species was therefore used to make some possible projections (Figure 4). Projections were made considering that “Arancio Luce G” is likely to be an azoic acid dye, and that the counter-ion is likely to be Na+ (as is usually the case for this class). Regarding the species, which seems to be a singly charged azoic dye based on the isotopic pattern observed, the *m*/*z* is likely to represent [M-Na]^−^, where M would represent the molecular mass and be equal to 384.2506 u. When this information was searched on chemical databases, a tentative possible assignment to Acid Orange 31 was made [37].

- Assignment of “Giallo Eliaminia RL”

Preliminary investigations into the identification of “Giallo Eliamina RL” were strongly based on the nomenclature. Several chemical databases listed Yellow Eliamina as a synonym for a variety of dyes: Direct Yellow 29, Direct Yellow 44, Direct Yellow 49, and Direct Yellow 50. Except for Direct Yellow 29, the other molecules share some features. Specifically, they are diazo structures with the presence of a central carbamide group. 

Mass spectral data of the “Giallo Eliamina RL” powder revealed several species present in the sample; however, a very intense peak corresponding to *m*/*z* 388.7797 at a retention time of 2.25 min was by far the most prominent. The species seems to be a singly charged azoic dye; the *m*/*z* is likely to represent [M-Na]^−^, where M would represent the molecular mass and be equal to 411.7695u. Molecular weight searches into the projected mass were performed but no yellow dyes were found to correspond.

- Assignment of “Giallo Italana 2G”

In the case of Giallo Italana 2G, preliminary studies were not indicative for identification. 

The mass spectral data for “Giallo Italana 2G” presented three *m*/*z* species, and by far the most intense was *m*/*z* 236.9883, which had a retention time of 3.35 min (Figure S1 in Supplementary Materials). The species, based on isotopic pattern observed, is a singly charged azoic dye; thus, the *m*/*z* is likely to represent [M-Na]^−^, where M would represent the molecular mass and be equal to 259.9781u. Molecular weight searches into the projected mass were performed but no yellow dyes were found to correspond. However, it is possible to infer from the Raman spectrum [9] that the spectral template for “Giallo Italana 2G” is likely to share structural details with Acid Orange 31.

- Assignment of “Giallo Luce Solido 2G”

Preliminary research on both the nomenclature and Raman spectra agreed that it was highly likely that that “Giallo Luce Solido 2G” powder was likely to be the dye Acid Yellow 11. From the interpretation of the Raman spectrum, whilst there were some spectral similarities between the fiber sample and the powder sample, several of the peaks did not correspond strongly [9]. Preliminary analyses considered that this may be due to the dyeing process, but also introduced the possibility that the fiber and powder may have different molecular structures despite sharing the same name.

**Powder:** The mass spectrometry analysis of the powder sample corroborated the prediction that the dye was Acid Yellow 11. The mass spectrometry results indicated the presence of a very intense peak corresponding to *m*/*z* 357.0572 between retention times 2.59–2.99 min (Figure S2). The *m*/*z* corresponds to the following species: [M-Na]-, where M is the mass of Acid Yellow 11 and is equal to 380.0555u. The spectrum of the “Giallo Luce Solido 2G” powder was therefore considered highly likely to be Acid Yellow 11 [38].

**Fiber:** For the fiber species, there is no significant peak present corresponding to the *m*/*z* 357.0572. This agrees with the preliminary Raman data in which the spectra did not appear to completely match with the powder spectrum despite some similarities [9]. This indicates that the powder and fiber samples named “Giallo Luce Solido 2G” are different molecular species despite having the same commercial names.

The fiber sample did however present a very intense chromatographic peak corresponding to an *m*/*z* 417.3234 at retention time 3.88 min. From this *m*/*z*, it is possible to draw several interpretations, but if it is considered likely that the fiber is an azoic acid (which the similarities in the Raman spectrum to the powdered sample would point towards) and that the counter-ion is likely to be Na+, some projections for possible masses can be put forward: the molecule, if singly charged, is characterized by a *m*/*z* ratio likely representative of [M-Na]-, and the molecular mass would likely be to be 440.3132 u. Molecular weight searches into the projected mass were performed but no yellow dyes were found to correspond.

- Assignment of “Giallo Novamina 2G”

Three molecules were proposed as possible identifications of the dye “Giallo Novamina 2G” from preliminary research on nomenclature: Acid Yellow 61, Acid Yellow 39 and Acid Yellow 25. Whilst no reference Raman spectra were available for Acid Yellow 61 and 39 for comparison, the Raman analysis on an analytical standard of Acid Yellow 25 performed in the laboratory was found to correspond strongly to the peaks of the “Giallo Novamina 2G” Raman spectrum.

Upon mass spectrometric analysis, an intense chromatographic peak was observed corresponding to *m*/*z* 526.0876 at retention time 2.86 min, which is exactly as observed for the Acid Yellow 25 analytical standard. The *m*/*z* corresponds to the following species: [M-Na]-, where M is the mass of Acid Yellow 25 with a sodium counter-ion and is equal to 549.0774u.

The chemical structure of “Giallo Novamina 2G” is therefore understood as highly likely to be that of Acid Yellow 25 [39].

- Assignment of “Rosso Amidonaftolo 2G”

The predictions proposed by the preliminary research on both the nomenclature of the dye “Rosso Amidonaftolo 2G” and the comparison of the Raman spectra obtained meant that Red 2G was predicted as a likely candidate for the identification of the molecular structure of both the powder and fiber samples.

This identification was corroborated by the presence of an intense chromatographic peak corresponding to the *m*/*z* 464.0233 at retention times 2.17 min for the powder (Figure S3) and 2.24 min for the fiber, which are close to those observed for the Red 2G standard. Furthermore, another the peak at *m*/*z* 358.9780 was observed, as reported in the literature [40]. 

Both the powder and fiber samples of Rosso Amidonaftolo 2G are hence identified as highly likely to be Red 2G [41].

- Assignment of “Tartrazina J”

The preliminary predictions for “Tartrazina J” were tartrazine (based on nomenclature; however, significant spectral differences were observed between the Raman spectra for these compounds) and Acid Yellow 17 (based on a very strongly similar Raman spectrum) [9,42].

For Acid Yellow 17, the expected *m*/*z* peak is likely to exist at *m*/*z* 251.6546, corresponding to the species [M-2Na]^2−^, where M is the mass of Acid Yellow 17 and is equal to 551.2888u. This peak was not present in the spectrum of Tartrazina J, and it was therefore inferred that the dye molecule is unlikely to correspond to Acid Yellow 17.

For tartrazine, there could be significant problems in detection with mass analysis owing to a triple charge on a very small molecule and the existence of the molecule in several states. In trials undertaken as part of this work, even an analytical standard of tartrazine was could not be detected in targeted analysis, so it is unlikely that it would be possible to detect the species in untargeted analysis—which is less sensitive for specific compounds. Alongside this, the Raman spectrum of “Tartrazina J” showed some significant spectral peaks that did not correspond to the peaks observed in the analytical standard of tartrazine.

However, one of the recorded *m*/*z* peaks in the literature for tartrazine is *m*/*z* 233.1, owing to the following species: [M-3Na+H]^+^ [43], and this peak was present in the mass spectrum of “Tartrazina J”, alongside another equally intense peak at *m*/*z* 228.9509 (Figure S4). Due to problems with analyzing Tartrazine, it was not possible to conclude whether tartrazine may have been present in the sample, but if it is present, it is possible that the dyed fiber may contain a mixture of dyes, which would account for the extra peaks on the Raman spectrum [9].

- Assignment of “Rosso Italana B”

Preliminary research on the nomenclature of the “Italana” dyes yielded no results. Raman spectral comparisons with databases indicated possible correlations with structures similar to Acid Red 26 [9]. Upon corroboration with the mass spectral data, however, this possible attribution was found to be unlikely, as the following predictions were made based on the only diagnostic peak that appeared on the spectra, which had an *m*/*z* of 236.9884 and a retention time of 3.36 min. If the species is a singly charged azoic dye, the *m*/*z* is likely to represent [M-Na]^−^, where M would represent the molecular mass and be equal to 259.9782 u. If the species is a doubly charged azoic dye, the *m*/*z* is likely to represent [M-2Na]^2−^, where M would represent the molecular mass and be equal to 519.9564u.

Molecular weight searches into the two projected masses were performed but no red dyes were found to correspond.

- Assignment of “Rosso Luce Solido BL” and “Rosso Italana R”

For both “Rosso Luce Solido BL” and “Rosso Italana R”, *m*/*z* peaks corresponding to the major peaks in the chromatogram were all present in a wide range of the spectra acquired from the whole set of the museum’s dyes and were hence not considered to be indicative of the dye compounds present in the samples. It is possible that, upon further analysis of the chromatograms in the laboratory, other chromatographic peaks may be identified, with more diagnostic *m*/*z* values. For “Rosso Italana R” in particular, a very broad peak was acquired from 1.45–2.41 min, which unfortunately includes the retention time of an extremely intense peak at *m*/*z* 360.3130, which appears in every spectrum. It is therefore likely that if the diagnostic peak elutes within this range that its signal may be overwhelmed by the intensity of the *m*/*z* 360.3130 peak in the mass spectrum and therefore not be visible. For both “Rosso Luce Solido BL” and “Rosso Italana R”, the tentative data obtained from preliminary predictions were not adequate for making any informed decision about the identification of dyes without a defined mass peak.

- Assignment of “Rosso Naftolo SJ”

Naphthol reds are a very common and significantly varied range of azoic acid dyes, so the name “Rosso Naftolo SJ” is not particularly indicative for obtaining preliminary predictions from. Unfortunately, no preliminary predictions were possible based on Raman comparisons [9].

This meant that the interpretation of the mass spectral data was approached only with the understanding that the dye was likely to be a species containing a naphthalene group. For interpretation of the chromatogram, the only intense peak considered likely to be indicative of the molecular structure of the compound was the peak recorded at a *m*/*z* of 200.9741 and retention time of 3.08 min. This was therefore used to make a tentative projection for the possible mass of the molecule as follows: if the species is a singly charged azoic dye, the *m*/*z* is likely to represent [M-Na]^−^, where M would represent the molecular mass and be equal to 223.9308u; if the species is a doubly charged azoic dye, the *m*/*z* is likely to represent [M-2Na]^2−^, where M would represent the molecular mass and be equal to 447.8616u.

Molecular weight searches into the two projected masses were performed but no red dyes were found to correspond. The authors contend that due to the likelihood that the dye molecule contains a naphthalene group (as suggested by the name) as well as at least one sulfonate group and one azo group (as is the case for the other dyes identified from the sample set), it is suspected that a molecule of mass 223.9308u is unlikely to correspond as these components have a cumulative mass > 223.9308u.

- Assignment of “Rosso Novamina 2G”

Preliminary research on the nomenclature of “Rosso Novamina 2G” found two related azoic acid dye species—however, a historical document [44] strongly indicated that “Rosso Novamina 2G” is likely to be Acid Orange 19 [45]. No Raman spectra were available in the literature for the standard for spectral comparisons to be performed, and the Raman spectrum of “Rosso Novamina 2G” was also very strongly affected by the signals of the wool compared to the other red dyes, meaning that only very few peaks were visible.

As such, the prediction was made solely on the nomenclature for this sample and it was predicted that if the sample corresponded to Acid Orange 19, which has a mass of 519.0535u, then the major *m*/*z* peak in the mass spectral data should correspond to the following species: [M-Na]^−^ = 496.0655. Indeed, an intense peak corresponding to this *m*/*z* was observed at a retention time of 2.88 min (Figure S5).

It is hence proposed that the identification of the dye “Rosso Novamina 2G” is likely to be Acid Orange 19.

- Assignment of “SEII Azoico Acido Pag”

For “SEII Azoico Acido Pag”, unlike the other samples, the label on the glass jar was simply handwritten. Due to this (alongside the fact that the naming format was very different to the others in the set), it was deemed likely that the label may have simply corresponded to an internal sample management system and not the commercial dye nomenclature. Research on the name found no bibliographic references, and a Raman spectrum of this sample was not obtained due to the spectrum being overwhelmed by fluorescence.

**Figure 5:**
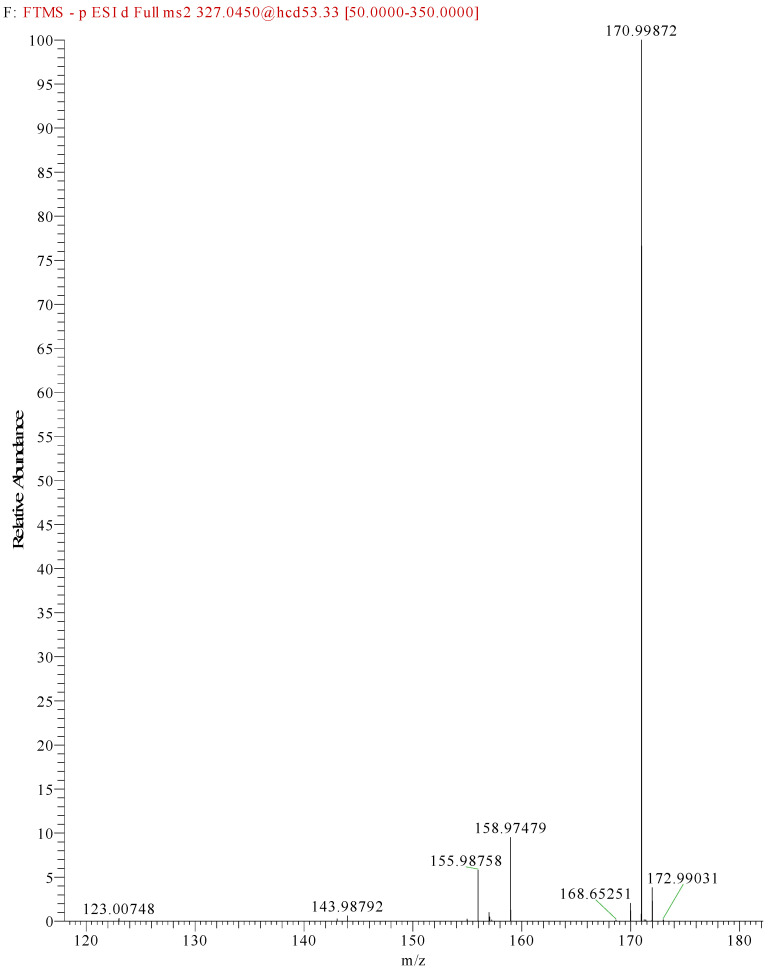
Fragmentation of peak at *m*/*z* 327.0450, 2.73 min. The two diagnostic fragments at *m*/*z* 170.9987 and 155.9875 are visible.

This meant that the mass spectrometry data were observed without any prior knowledge or corroborative data about the possible molecular structure and hence were difficult to interpret. There were several very high-intensity *m*/*z* peaks observed in the data. A fairly intense chromatographic peak was observed corresponding to the *m*/*z* 231.5072 associated with Red 2G at a retention time of 2.14 (as observed with the standard), as well as intense peaks at *m*/*z* 217.01257, 253.1350, 327.0452, 355.0767 and 577.4851. The peak at *m*/*z* 327.0452—recorded at a retention time of 2.68 min—was by far the highest peak observed; however, all peaks were of a significant magnitude, indicating that it is possible that the sample is a mixture. This peak showed two diagnostic fragments at *m*/*z* 170.9987 and 155.98758 (Figure 5); for this reason, taking into account the literature data [46], it was identified as Acid Orange 7 [47]. 

## 3. Materials and Methods

### 3.1. Solvents and Reagents

High-purity analytical standards of Congo Red and Red 2G were purchased from Sigma Aldrich. A ≥ 40% purity standard of Acid Yellow 25 was also purchased from Sigma Aldrich. Solvents, acids and bases were purchased from Sigma Aldrich and used without further purification. Na_2_EDTA∙2H_2_O was purchased from Carlo Erba while TBAB and other salts were purchased from Sigma Aldrich.

### 3.2. Development of Clean Up for Synthetic Dyes: dLLME

Development of the cleanup protocol was performed on a mixed reference sample containing 500 ppb of Acid Yellow 25, Congo Red, and Red 2G. The analytes were dissolved to 500 ppm and diluted to 100 ppm in Millipore water and the final dilution to 500 ppb was then performed in methanol. All experiments were repeated three times and each replicate was analyzed twice using mass spectrometry to obtain an average.

#### 3.2.1. Evaluation of Disperser for Synthetic Dyes

The dLLME method was adapted from a protocol outlined by Faraji et al. [24] for the determination of azo dyes from ice cream samples. Chloroform was used as the extracting solvent for the tests and methanol, isopropanol, acetonitrile and acetone were all trialed as dispersers.

The disperser trials were carried out as follows: 500 μL of 100 ppb mixed standard was placed together in a tube. The sample was then made up to 5 mL with Millipore water, and 150 μL previously made up 2M tetra-n-butylammonium bromide (TBAB) in water was added as the ion pair reagent. The tube was lightly swirled to ensure homogeneity. Quantities of 750 μL of the desired disperser and 100 μL of chloroform were then drawn up into a syringe and injected rapidly into the aqueous phase, forming a cloudy solution. The mixture was then vortexed for 10 s and sonicated for 10 min before undergoing 5 min of centrifugation at 4200 rpm. The bottom organic layer was removed with a syringe and placed in a vial where it was dried under N_2_ flow. The extract was reconstituted with 100 μL methanol for analysis using HPLC-MS.

#### 3.2.2. Trial of the Complete Analytical Protocol for Synthetic Dyes

After the dLLME procedure was set up, the chosen method was paired with the initial extraction protocol to ensure functionality of the whole protocol when joined together. The initial extraction method was exactly as described in the literature for natural dyes in Serafini et al. 2017 [21]. After that, the dLLME protocol was applied, employing methanol as the disperser, as described above.

#### 3.2.3. Analysis of Historical Textile and Powder Samples

The fiber samples were extracted using the method described above. Specifically, the fiber sample was placed in a vial containing 4.4 mg NaCl, 0.8 mL 30% NH_3_ and 0.8 mL 1 mM Na_2_EDTA. The samples were left in the extraction mixture and covered in aluminum foil for 2 days and left to extract at room temperature. The solution was then pipetted out. The sample was then placed under N_2_ flow to facilitate the evaporation of the ammonia, and this was performed until a neutral solution was obtained. 

The neutral solution was then placed in a tube and made up to 5 mL with Millipore water to which 150 µL 2M TBAB was added. The tube was lightly swirled to ensure homogeneity. Quantities of 750 µL methanol and 100 µL chloroform were then drawn up into a syringe and injected rapidly into the aqueous phase, forming a cloudy solution. The mixture was then vortexed and sonicated for 10 min before undergoing 5 min of centrifugation at 4200 rpm. The bottom organic layer was removed and placed in vial, where it was dried under N_2_ flow.

For the powder samples, it was not necessary to perform the initial extraction step and therefore the samples were dissolved in 5 ml water in a tube and the dLLME protocol was then performed directly on the sample.

HPLC-HRMS was then carried out using untargeted analysis by following the instrumental setup reported below.

### 3.3. HPLC-MS Analyses

#### 3.3.1. Targeted HPLC-MS Analyses

Recovery analysis during method development was carried out using the results from targeted HPLC-MS. For the chromatographic analysis, a Series 200 Perkin Elmer micro-LC system equipped with an autosampler was used. The system was coupled to a PE-Sciex API 2000 triple quadrupole mass spectrometer equipped with a TurboIon-Spray ionization source, operating in negative ionization mode. The columns tested were a Kinetex XB-C18 2.6 μm core–shell particle column and a Luna-C18 5 μm column, and the Luna-C18 was chosen. The system was used in SIM. To individuate the best chromatographic conditions for synthetic dyes, three trials were performed on the mobile phases:

i. Phase A: 0.1% formic acid in acetonitrile; Phase B: 0.1% formic acid in water;
ii. Phase A: 5 mM ammonium acetate in acetonitrile; Phase B: 5 mM ammonium acetate in water;
iii. Phase A: methanol; Phase B: 5 mM ammonium acetate in water.

The mobile phases chosen were methanol and 5 mM ammonium acetate in Millipore water in a gradient elution, as shown in Table 2.

The SIM targets are listed below, in Table 3:

Recoveries were calculated by performing peak area integrations with Sciex Analyst software, and these areas were compared to results from the original standards in Microsoft Excel.

#### 3.3.2. Untargeted HPLC-MS Analyses

Untargeted mass spectrometric data of the unknown museum samples were acquired using a Thermo Fisher Scientific DionexTM UltiMateTM 3000 (RSLC) UHPLC system equipped with an RS autosampler and coupled with a high-resolution Q-Exactive Orbitrap mass spectrometer equipped with a heated electrospray ionization source (H-ESI).

The H-ESI source operated in negative ionization mode with tuning parameters set at sheath gas flow rate (nitrogen) = 45 units, auxiliary gas flow rate (nitrogen) = 20 units, spray voltage = −3.00 kV, capillary temperature = 350 °C, source temperature = 350 °C. MS experiments were carried out in full scan–data dependent acquisition mode (Full-dds). A full scan was conducted with a scan range between 100 and 800 *m*/*z* with a resolution of 70,000 FWHM; automatic gain control (AGC) was 1 × 106, maximum injection time was 100 ms. For MS/MS experiments, resolution was 17,500 FWHM, AGC was 5 × 10^6^, maximum injection time was 80 ms, loop count and TopN were 5, isolation window was set to 2.0 *m*/*z*, the fixed first mass was 50 *m*/*z*. Minimum AGC target was 8 × 10^3^ and the intensity threshold was 1 × 10^6^. The dissociation of molecular ions was induced in a high-energy collision cell (HCD) by means of nitrogen; simultaneous experiments were conducted at three different normalized collision energies: 10, 30 and 50%.

The Luna-C18 column used for the setting of the methods and the mobile phase conditions individuated for the method development were used for analysis on historical samples.

### 3.4. Sampling from ACNA Collection

As mentioned in the introduction, the collection partially studied in this work comes from the Museum of Chemistry, located within Sapienza University of Rome’s Department of Chemistry, which holds an extensive collection of early synthetic dyes from several different dye companies. ACNA industry, an acronym for Azienda Coloranti Nazionali e Affini (ACNA), was an Italian chemical company active from 1882 until 1999 [6]. The company was extremely controversial throughout the entirety of its history. It was first founded as an explosives factory that operated under the name Dinamitificio Barbieri and subsequently the Italian Society of Explosive Products. The company later moved away from the production of explosives and retooled as a colorant manufacturer after being acquired by Italgas in 1925. The ACNA received significant investment from the fascist regime in the years immediately following 1925, to promote Italian manufacturing industries, and its name was once again changed, this time to the Associated National Chemistry Companies—at which point it obtained the acronym ACNA. It was then acquired by the larger companies IG Farben and Montecatini, who gave it its final name: Azienda Coloranti Nazionali e Affini [48]. Under these companies, the ACNA manufactured colorants, working until 1999, during which time their production caused extensive pollution of the surrounding areas, inflicting severe damage to both the environment and the health of nearby residents [48].

Sampling of the ACNA Museum’s dye collection was carried out in July 2020 after a preliminary viewing of the collection. The preliminary visit was used to take photographs and note commercial names of dyes in the collection, which were then researched in the subsequent months to identify possible assignments for the dyes based on the nomenclature. Fibers (less than 1 mg) and powdered samples (about 1 mg) were then selected as the case study for this research.

## 4. Conclusions

The primary aim of this work was the development and application of a novel extraction strategy, based on the ammonia–EDTA protocol, adding a novel cleanup protocol for the purification and enrichment of the analytes for the analysis of synthetic dyes from historical and artistic matrices. As a secondary aim, the protocol developed for synthetic dyes was applied to a real case study of historical synthetic dyes from the Museum of Chemistry, Sapienza University to verify the effectiveness of this protocol in historical sample analyses. This research adapted a methodology from the analytical science of food and successfully developed a protocol well-suited to cultural heritage. For the dLLME protocol, the best results were obtained with the use of methanol as the disperser solvent with chloroform as the extracting solvent. 

One of the main benefits of this application is that it allows for synthetic dyes to be extracted alongside natural dyes in a single extraction step, with the ammonia extraction protocol, and the best-suited dLLME protocol can be employed to purify the analytes. In this way, both types of dye now only require the acquisition of a single sample instead of two, minimizing the destructiveness of analysis.

For application to synthetic dye samples from the ACNA industrial collection, the application of the novel analytical protocol for the extraction and preconcentration of synthetic dyes was effective for this research, and it can therefore be considered a significant contribution to the study of synthetically dyed artefacts. Furthermore, the results obtained confirmed the importance of scientific study in improving understanding of naming conventions in the context of the synthetic dye collections. In ACNA dyes, particularly interesting was the case of “Giallo Italana 2G”, where it appears that, despite being labelled with the same name and being from the same company, the dyed fiber and powdered sample are likely to have different chemical structures. In contrast, in “Rosso Amidonaftolo 2G”, the chemical structure was consistent between the powder sample and the dyed wool. This is a strong exemplification of the complexity of the study of synthetic dye collections—where, even within a single company, naming conventions can vary widely. 

The case study also perfectly illustrates the benefits and necessity of a multi-analytical approach to the analysis of unknown artefacts. In some cases, Raman spectroscopy was highly indicative of the likely assignment of the structure, but when compared with the mass spectral results, the preliminary assignment was shown to be incorrect. This was illustrated with the “Tartrazina J” sample, which appeared strongly similar to the spectrum for Acid Yellow 17, but the expected *m*/*z* was not observed in the mass spectrum. Contrastingly, for some samples, the indicative nature of the Raman meant that mass spectra could be rapidly interpreted to corroborate the data, for example, with “Rosso Amidonaftolo 2G”. In other cases, such as for “Arancio Luce G”, the preliminary data were not indicative of a particular compound but could be used to corroborate the data obtained from the mass spectrum. 

Moreover, the preliminary investigations using Raman analyses once again demonstrated the importance of a multi-analytical approach, which meant that several samples from the collection could be analyzed with only a very small textile sample.

It is also hoped that further studies will be conducted in the future, and hopefully provide more indicative data for the unidentified samples, thus implementing the database of such dyes. 

## Figures and Tables

**Table 1:** A summary table of evidences found and/or hypothesized for each sample.

Sample	Possible Match or Possible Chemical Features	Chemical Structure
Arancio Luce G	Acid Orange 31	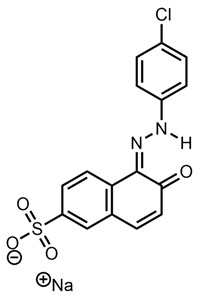
Giallo Eliaminia RL	Further studies ongoing	
Giallo Italana 2G	Structural similarities with Acid Orange 31	
Giallo Luce Solido 2G—powder	Acid Yellow 11	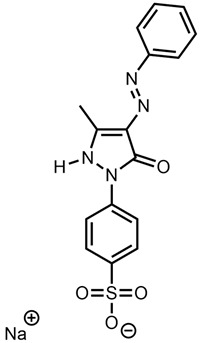
Giallo Luce Solido 2G—fiber	Further studies ongoing	
Giallo Novamina 2G	Acid Yellow 25	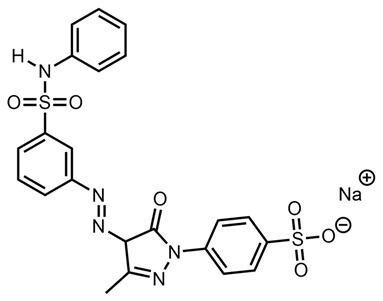
Rosso Amidonaftolo 2G	Red 2G	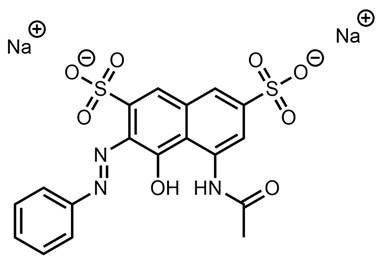
Tartrazine J	Further studies ongoing	
Rosso Italana B	Further studies ongoing	
Rosso Luce Solido BL; Rosso Italana R	Further studies ongoing	
Rosso Naftolo SJ	A naphthalene group (as suggested by the name) as well as at least one sulfonate group and one azo group	
Rosso Novamina 2G	Acid Orange 19	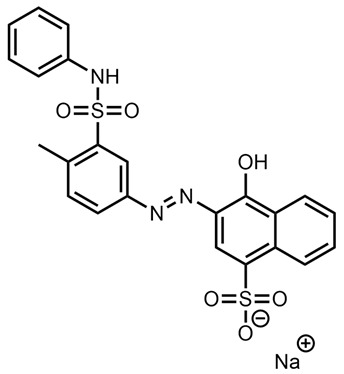
SEII Azoico Acido Pag	Acid Orange 7	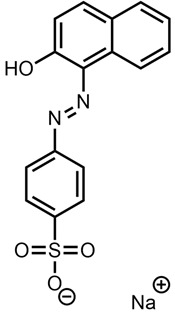

**Table 2:** Gradient elution for HPLC-MS analyses.

Time (min)	Phase A (MeOH)	Phase B (5 mM Ammonium Acetate in H_2_O)
0.0	0%	100%
3.0	100%	0%
4.5	100%	0%
5.0	0%	100%
7.0	0%	100%

**Table 3:** Synthetic dye standard mass spectrometry targets.

Dye	Exact Mass	Parent Ion (*m*/*z*)	DP (V)	FP (V)	EP (V)	CEP (V)
Acid Yellow 25	549.5537	526.2	−101	−140	−9	−29.66
Congo Red	696.6622	325.3	−38	−365	−9	−24.79
Red 2G	509.4200	231.5	−15	−326	−7	−22.52

## Data Availability

Not applicable.

## Supplementary Materials

The following supporting information can be downloaded at: https://www.mdpi.com/article/10.3390/molecules28145331/s1, Figure S1: Diagnostic peak at 236.9883 from Giallo Italana 2G; Figure S2: Chromatograms of Giallo luce solido 2G, powder, with the diagnostic peak at 357.0572 at 2.59–2.99 min; Figure S3: Diagnostic peak at 464.0233 from Rosso Amidonaftolo-powder. Figure S4. Chromatograms of Tartrazine J, fiber; Figure S5: Chromatograms of Rosso Novamina 2G, fiber, with the diagnostic peak at *m*/*z* 496.0655, 2.88 min.
