# Supplementary material for: Inside the History of Italian Coloring Industries: An Investigation of ACNA Dyes through a Novel Analytical Protocol for Synthetic Dye Extraction and Characterization
Source: Molecules. 2023 Jul 11;28(14):5331. doi: 10.3390/molecules28145331 (PMC10386332; doi:10.3390/molecules28145331)
Supplement: Supplementary file 1 [file molecules-28-05331-s001.zip › molecules-2400574-supplementary.pdf]

Inside the history of Italian coloring Industries: an investigation into the ACNA dyes through a novel analytical protocol for the synthetic dyes extraction and characterization

Ilaria Serafini[1*], Kathryn Raeburn McClure[1], Alessandro Ciccola[1], Flaminia Vincenti[1], Adele Bosi[1,2], Greta Peruzzi[1], Camilla Montesano[1], Manuel Sergi[1], Gabriele Favero[3], Roberta Curini[1]

[1] Dept. Chemistry, Sapienza University of Rome, P. le Aldo Moro 5, 00185, Rome
[2] Dept. Earth Sciences, Sapienza University of Rome, P. le Aldo Moro 5, 00185, Rome
[3] Dept. of Environmental Biology, Sapienza University of Rome, P. le Aldo Moro 5, 00185, Rome Italy

* Correspondence: ilaria.serafini@uniroma1.it

Supplementary Materials

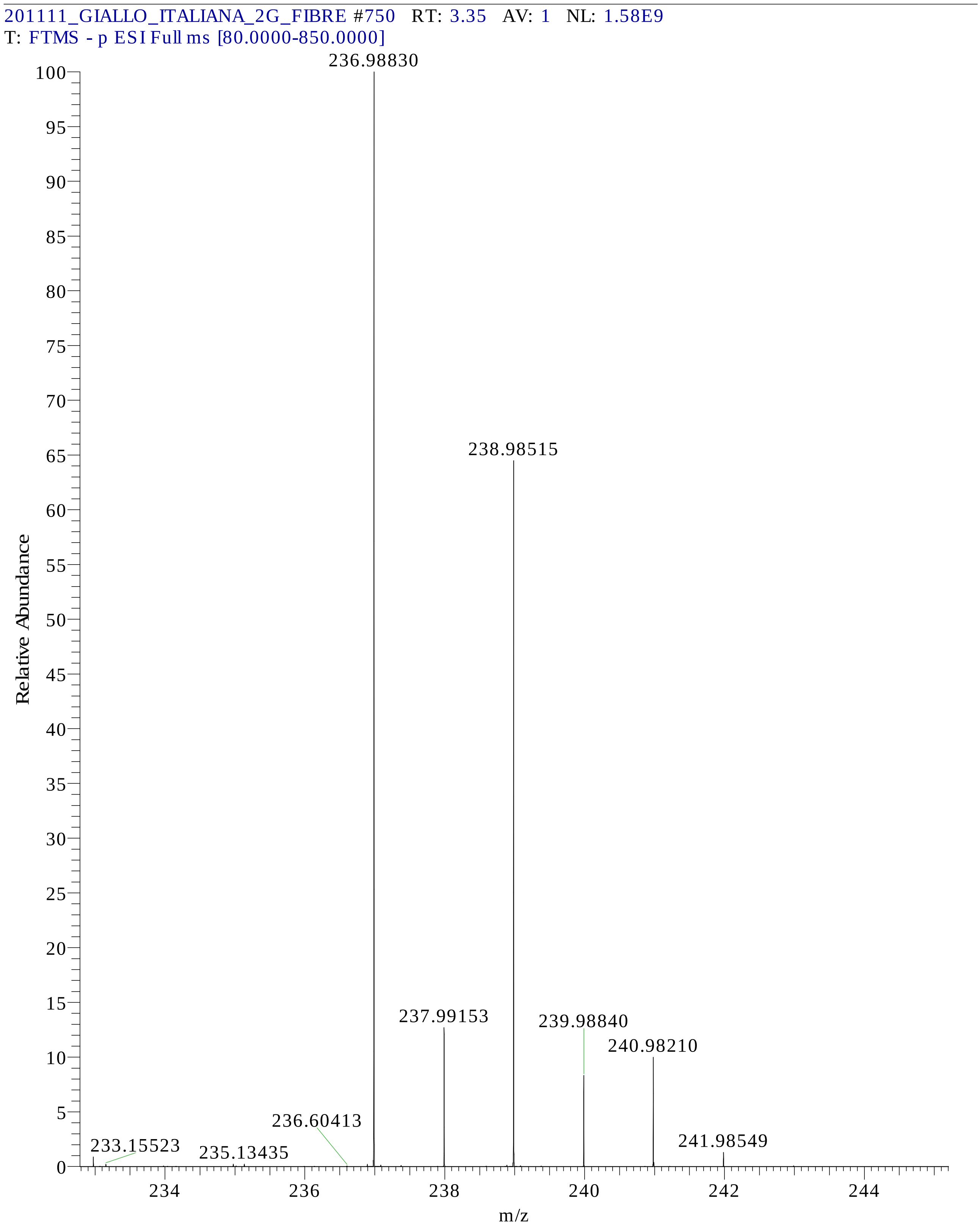

Figure S 1. Diagnostic peak at 236.9883 from Giallo Italana 2G.

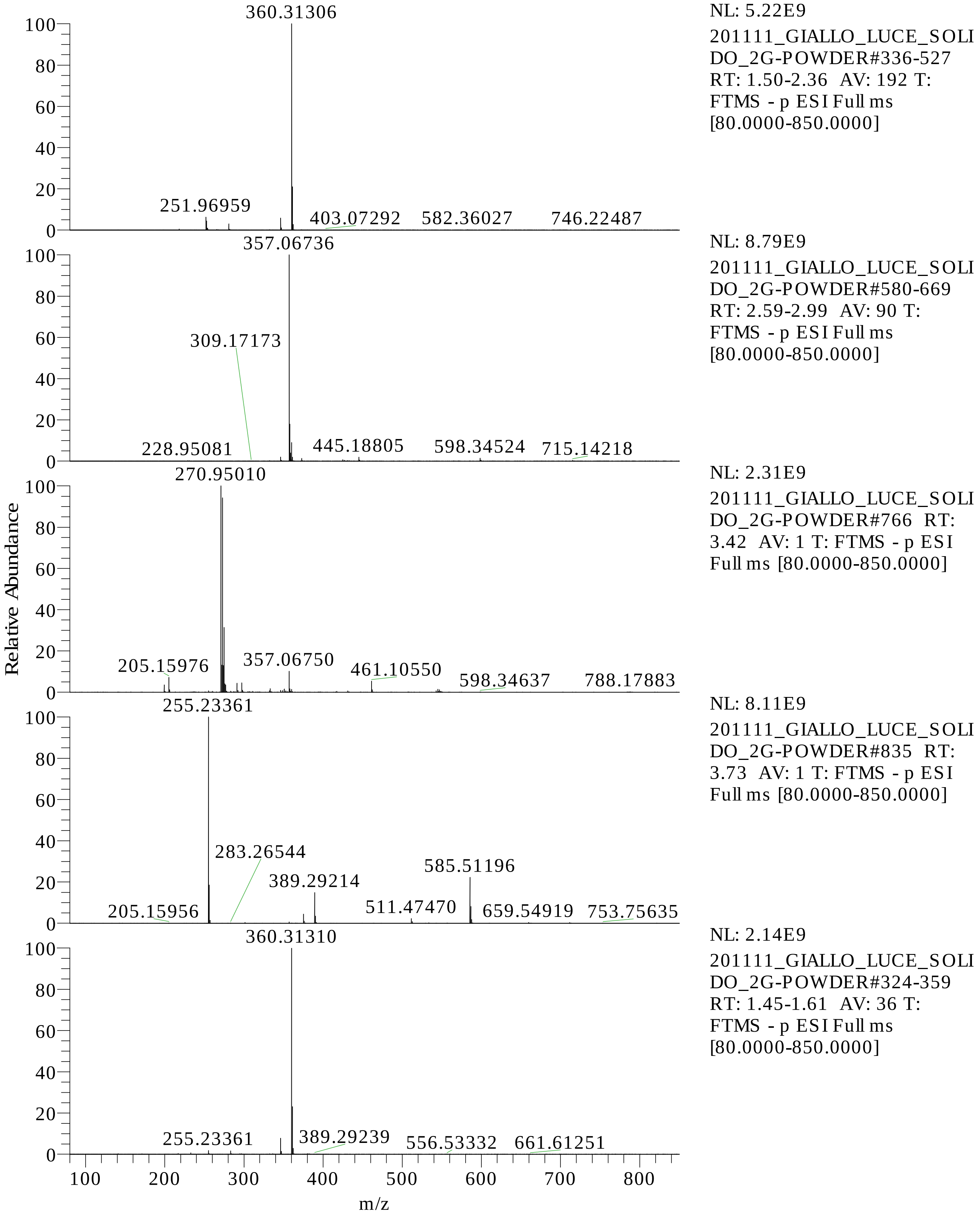

Figure S 2. Chromatograms of Giallo luce solido 2G, powder, with the diagnostic peak at 357.0572 at 2.59-2.99 min.

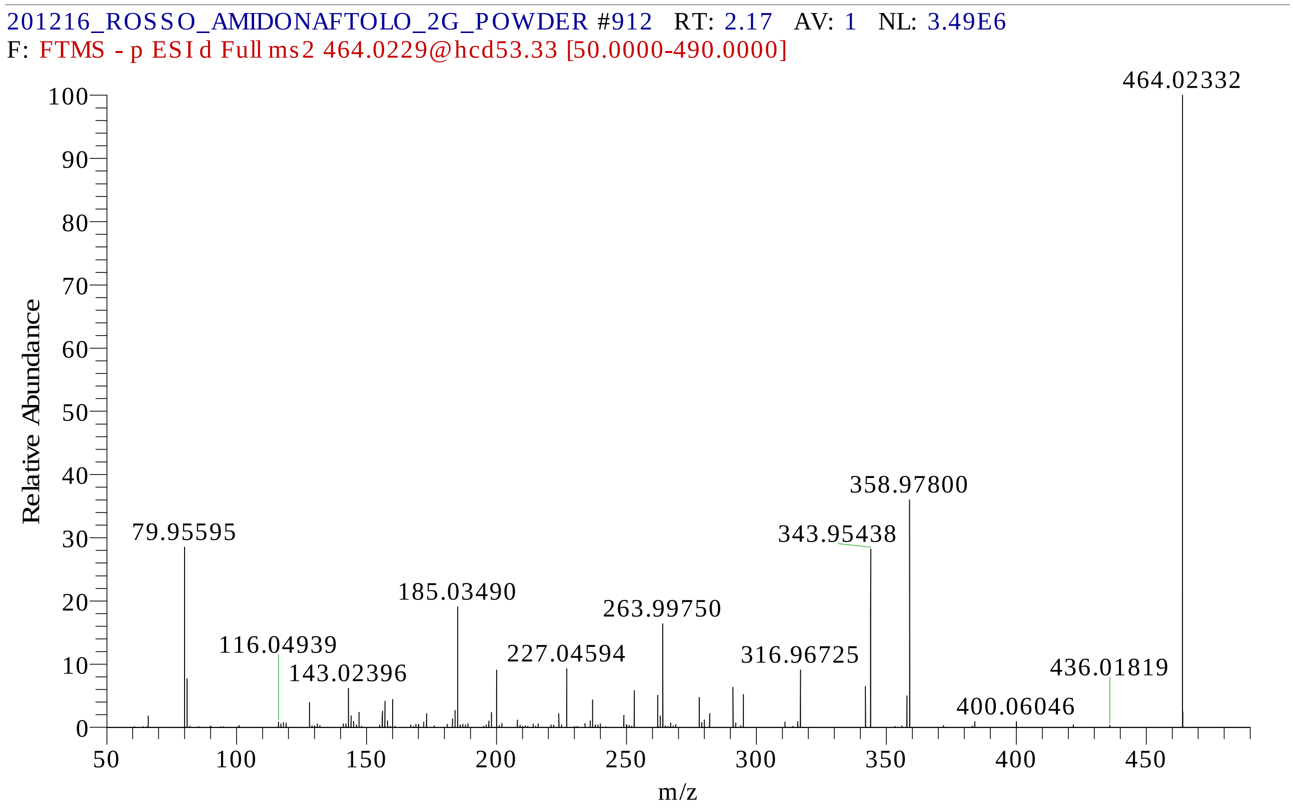

Figure S 3. Diagnostic peak at 464.0233 from Rosso Amidonaftolo-powder.

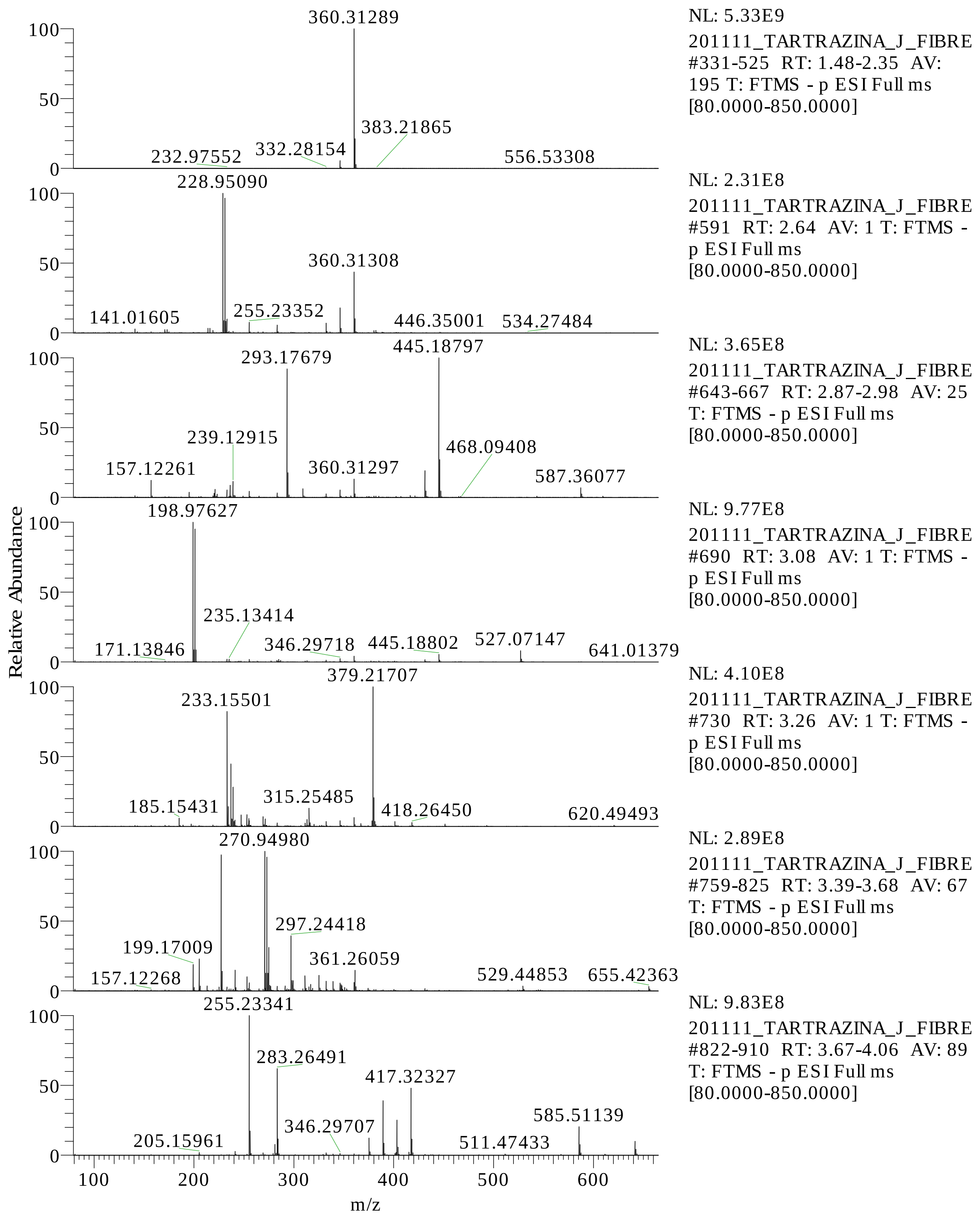

Figure S 4. Chromatograms of Tartrazine J, fiber.

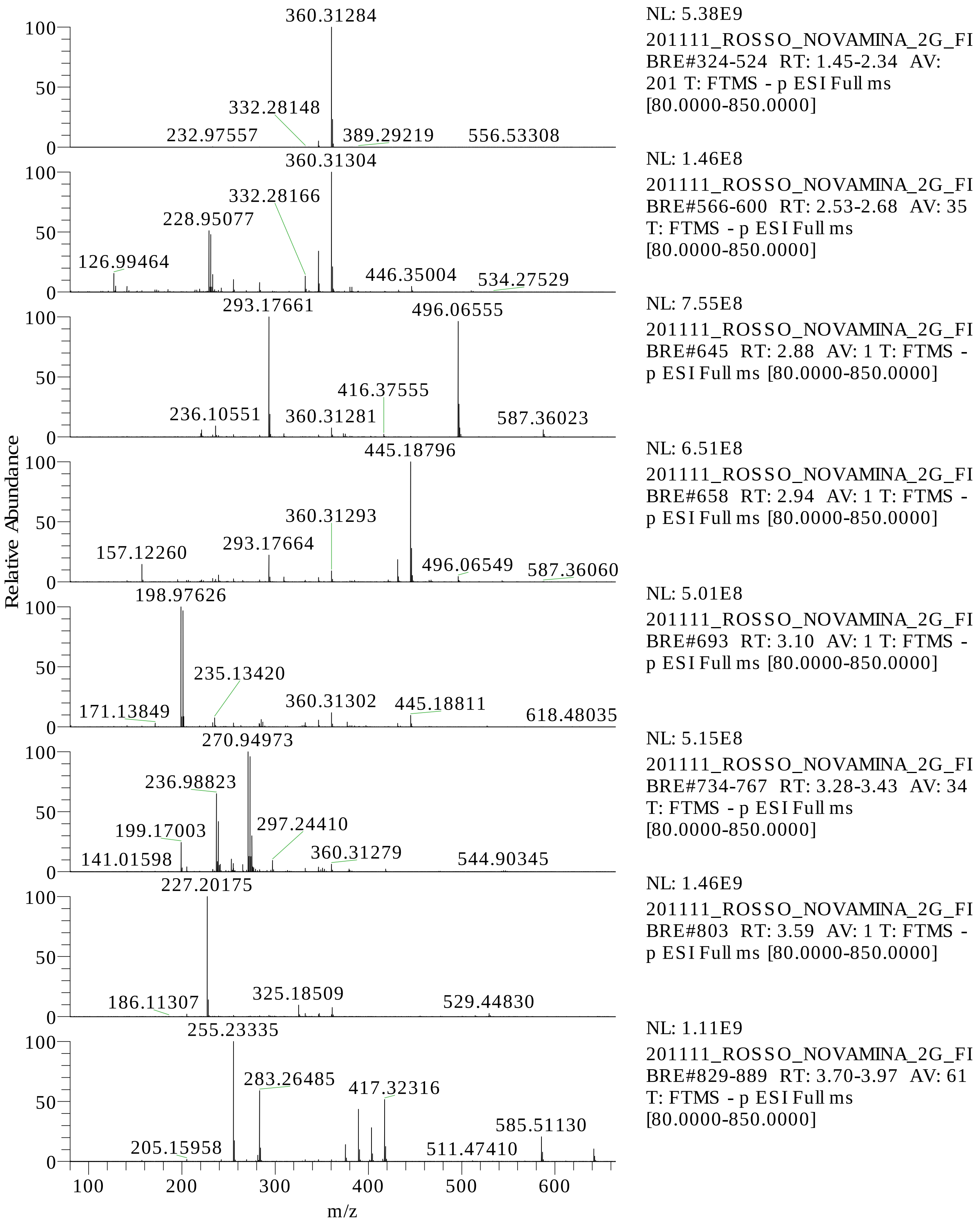

Figure S 5. Chromatograms of Rosso Novamina 2G, fiber, with the diagnostic peak at m/z 496.0655, 2.88 min.
